# Experimental Realization of a Reflections-Free Compact Delay Line Based on a Photonic Topological Insulator

**DOI:** 10.1038/srep28453

**Published:** 2016-06-27

**Authors:** Kueifu Lai, Tsuhsuang Ma, Xiao Bo, Steven Anlage, Gennady Shvets

**Affiliations:** 1 Department of Physics, The University of Texas at Austin, Austin, Texas 78712, USA; 2 Center for Nanophysics and Advanced Materials, Department of Physics, University of Maryland, College Park, Maryland 20742-4111, USA; 3 Department of Electrical and Computer Engineering, University of Maryland, College Park, Maryland 20742-3285, USA

## Abstract

Electromagnetic (EM) waves propagating through an inhomogeneous medium are generally scattered whenever the medium’s electromagnetic properties change on the scale of a single wavelength. This fundamental phenomenon constrains how optical structures are designed and interfaced with each other. Recent theoretical work indicates that electromagnetic structures collectively known as photonic topological insulators (PTIs) can be employed to overcome this fundamental limitation, thereby paving the way for ultra-compact photonic structures that no longer have to be wavelength-scale smooth. Here we present the first experimental demonstration of a photonic delay line based on topologically protected surface electromagnetic waves (TPSWs) between two PTIs which are the EM counterparts of the quantum spin-Hall topological insulators in condensed matter. Unlike conventional guided EM waves that do not benefit from topological protection, TPSWs are shown to experience multi-wavelength reflection-free time delays when detoured around sharply-curved paths, thus offering a unique paradigm for compact and efficient wave buffers and other devices.

The existence of localized TPSWs at the PTI’s^1^,^2^,^3^,^4^,^5^,^6^,^7^,^8^,^9^,^10^,^11^,^12^,^13^ edge^6^,^7^,^10^, or at an interface between two PTIs with different electromagnetic properties^1^,^3^,^11^,^12^,^14^, holds great promise for photonic applications. Their scattering-free propagation along sharply-curved paths^14^ opens exciting opportunities across the electromagnetic spectrum, including optical isolators^15^,^16^ multiple-input multiple-output communications systems^17^, and topologically robust broadband optical buffers and time delay lines^18^,^19^. Remarkably, while the latter set of applications was the original motivation^6^ for PTI development, an experimental demonstration of such functionality has been elusive. For example, in one successful implementation of topologically protected edge transport that utilized an ensemble of high-Q resonators^7^, statistical properties of time delays were measured^20^. However, the combination of finite disorder and sharp resonances makes the development of a single-channel delay line in a given photonic structure extremely challenging.

An alternative PTI platform emulates the quantum spin Hall (QSH)^21^,^22^,^23^,^24^ effect by introducing a photonic analog of spin-orbital interaction using bianisotropic metamaterials^11^, as well as uniaxial^12^ or bianisotropic^14^ metawaveguides. Unlike nonreciprocal PTIs^3^,^4^,^5^, it does not require an external magnetic field or ferromagnetic materials. The spin degree of freedom can then be interpreted as the phase relationship between transverse electric (TE) and magnetic (TM) modes of the metamaterial—in-phase for the spin-up and out-of-phase for the spin-down states. In this Letter, an interface between two QSH PTIs is used to experimentally demonstrate a single-channel topologically protected delay line that employs only the edge modes and is not influenced by the bulk modes. Strong suppression of the bulk modes with respect to the edge modes is crucial because the former are strongly affected by lattice disorder and are subject to localization-based transport^25^ that affects the delay time^20^.

The specific platform used in this work is shown in Fig. 1a. The QSH PTI is comprised of the parallel-plate metal waveguide sandwiching a periodically arranged hexagonal array of metallic cylinders attached to one of the two metal plates and separated by a finite gap from the opposite plate. The simulated photonic band structure (PBS) of the PTI shown in Fig. 1b (see the caption and Methods for the physical dimensions, and the details of numerical simulations and measurements) reveals a complete topological band gap (gray-shaded area) of the bulk PTI. The bandgap was demonstrated by measuring a 30 dB transmission drop in the 
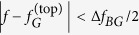
 frequency range (black line in Fig. 1c: 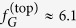 GHz and 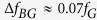) when all rods are attached to the top plate. A second, *topologically trivial* bandgap centered at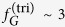 GHz (not shown), was also observed, and will be discussed later when comparing the topologically protected and trivial guided modes. Ohmic losses in the appropriately scaled PTIs are negligible for the frequencies as high as THz (see Methods), where there is growing interest in free-space wireless communications^26^ applications.

The topological spin-Chern index^27^ of the electromagnetic modes propagating below the bandgap changes sign^14^ when the rods are re-attached from the top to the bottom plate (see SI, section S1). Therefore, the Chern number is reversed across the wall between two QSH PTIs domains (“claddings”) with the rods attached to the opposite plates as shown in Fig. 1a. Such topological waveguide is expected^3^ to support four TPSWs plotted in Fig. 1b as red lines: two spin-up states propagating in the forward, and two spin-down states propagating in the backward directions (see section S2 of the SI). In the absence of spin-flipping perturbations, backscattering is prohibited for the TPSWs. Their existence across the entire bandgap is experimentally demonstrated (Fig. 1c) by measuring the ~30 *dB* transmission enhancement (red line) over that through the bulk PTI (black line). Therefore, the electromagnetic waves excited by the launching antenna inside the bandgap do not evanescently tunnel through the PTI’s bulk. Instead, they couple to the surface mode and propagate unimpeded towards the probe antenna. The spatial localization of the surface mode to a small fraction of the wavelength on either side of the interface is established by mapping the field profile in the *y*-direction (Fig. 2a). To our knowledge, this is the first experimental evidence of the wavelength-scale confinement of a surface wave propagating at the interface between two PTIs.

Finally, we demonstrate the topological protection of the surface wave by experimentally observing its most important physical property: that reflection-free energy flow can occur despite encountering a broad class of possible lattice defects along its propagation path that maintain spin-degeneracy^11^,^12^ and preserve the spin DOF (section S3 of the SI). Within this class falls the detour defect shown in the inset of Fig. 2b, where the rods are re-attached to the opposite plate so as to bend the interface between PTIs. The defect contains four 120° bends, each of which is capable of reflecting most of the incident surface wave in the absence of topological protection. As we show below, the addition of the defect creates a reflection-free single-channel delay line. The topological protection is apparent from Fig. 2b, where the transmission spectra along the uninterrupted interface (red line) and the same interface interrupted by a detour-type defect (green line) are plotted as a function of frequency. Outside of the bandgap (e.g., at the frequencies marked by black arrows) the transmission is reduced by almost an order of magnitude because the defect blocks the propagating bulk modes from the receiving antenna. Inside the bandgap, however, the forward-propagating spin-up TPSW flows around the defect (Fig. 2c) because the defect does not flip the spin, and no back-reflection is allowed. The almost negligible ~1 dB decrease in transmission (red arrows) serves as a clear experimental signature of topologically robust transport. Note that, while scattering against a broad class of defects can also be suppressed using electromagnetic cloaks^28^,^29^, such structures need to be tailored for specific defects and often require high-index dielectrics.

The uniqueness of topological protection of guided waves is underscored by comparing the topological waveguide described above with a topologically-trivial one, which is formed by removing one row of cylinders. The resulting waveguide (orange box inset in Fig. 3b) supports topologically trivial guided waves (TTGWs) that are spectrally located (orange curve in Fig. 3a) inside the topologically trivial band gap of the claddings centered at 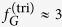 GHz (gray area). To emulate a time-delay line, we insert a large sharply-edged scattering defect (blue box inset of Fig. 3b) into the path of the TTGW. While the defect is almost identical to the one inserted into the path of the TPSW, its effect on the TTGW is dramatically different. For most frequencies, the measured transmission plotted in Fig. 3b (blue curve) is more than an order of magnitude lower than in the absence of the defect. High narrow-band transmission and tight spatial confinement (Fig. 3c) due to resonant tunneling^30^,^31^ and multi-bounce backscattering only occurs at the three Fabry-Pérot (FP) resonances of the defect. The absence of such FP resonances for the TPSWs observed from Fig. 2b is the experimental proof of their topological protection against backscattering.

These qualitative differences between topologically protected and trivial photon transport mechanisms motivates the usage of TPSW-based broadband delay lines. For example, a detour-type lattice defect shown in Fig. 2b introduces a time delay^32^


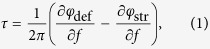


where *φ*_str_(*f* ) and *φ*_def_(*f*  ) are the phases of the transmitted EM waves for the straight and defect-interrupted interfaces. The time delay can be indefinitely increased by stacking multiple detour defects (Fig. 4). For example, two detours produce twice the delay time for the transmitted TPSWs, with no decrease in the bandwidth (Fig. 4a) because of the lack of interaction^18^ between adjacent defects due to topological protection. On the contrary, using two detour-type defects in a topologically trivial waveguide reduces the operational bandwidth (Fig. 4b) for TTGWs.

The key advantage of topologically protected delay lines is their compactness: TPSWs can make sharp turns into tightly-packed phase-delaying detours without any backscattering. By loading a straight PTI interface (which plays the role of a bus waveguide^18^ for the propagating TPSWs) with a sequence of phase-delay defects (Fig. 4, top), nearly arbitrary phase profiles *ϕ*_def_(*x*, *f*  ) can be generated and subsequently used in a variety of applications, including frequency-division multiplexing for free-space wireless communications^26^ and terahertz wave generation^33^. The detour defect shown in Fig. 2b,c can be viewed as a building block for these applications.

The experimentally measured phase and time delays of the transmitted TPSWs are plotted in Fig. 5a for the straight and defect-interrupted interfaces. Note that *τ*(*f* ) > 0 everywhere inside the gap, although considerable fluctuations are observed at the edges of the gap, where the amplitude of the transmitted TPSW is reduced due to incomplete topological protection. However, for most frequencies the time delay is spectrally flat. In contrast, without topological protection, the delay time of TTGWs is a rapidly-varying sign-changing^20^ function of the frequency inside the topologically trivial bandgap (Fig. 5b). These experimental results constitute the first step in building a multi-stage broadband topologically protected delay line capable of buffering multiple electromagnetic pulses.

In conclusion, we experimentally realized in the microwave frequency range a delay line based on a quantum spin Hall photonic topological insulator. Topological protection of localized surface waves between two PTIs resulted in reflectionless spectrally uniform time delays of several wave periods that were induced by a compact sharply-edged detour-type defect. Because such defects can be of nearly arbitrary shapes and sizes, we anticipate that novel geometries for compact wave buffers and delay lines utilizing topological photonic transport will emerge across the electromagnetic spectrum, from micro- to infrared waves. The introduced paradigm of an interface between two PTIs directed along a curved pathway will also enable creating near-arbitrary EM phase distributions *ϕ*(*x*, *y*, *f* ) in the plane of a waveguide. By creating slit openings in the waveguide’s wall, such phase distributions can be translated into complex frequency-dependent far-field patterns that can be utilized for frequency demultiplexing. Dynamic reconfiguration of the delay paths by rapid microelectromechanical displacement of the rods is also envisioned.

## Methods

### Transmission Measurements and Data Processing

Transmission measurement is performed with a linear dipole antenna as the feeding source which is inserted into the photonic structure through a small hole drilled through the top metallic cladding. When TPSWs are launched by such feeding source placed at the *x* = *x*_0_ location, the two spin-up waves (with positive and negative refractive indexes) are launched in the forward direction toward the receiving probe. The receiving probe is also a linear dipole antenna which is placed on the outside at the end of the structure. We use a 2-port VNA (Agilent E5071C) to extract the amplitude and phase of the transmitted EM waves from the measured raw |*S*_12_|^2^(*y*, *f*; *x*_0_) spectra as described below. The spectra are smoothed with a 50 MHz window to suppress spectral contribution of VNA cables.

The subsequent data analysis aims at smoothing the interference pattern produced by the superposition of the positive and negative index spin-up TPSWs. The spatial periodicity of the resulting intensity pattern is *P*_*x*_ = 3*a* along the interface separating the two PTIs. The following averaging treatment is used to suppress this beat pattern and to clarify the intensity distribution shown in Fig. 2: every transmission curve of the TPSWs is the average of 6 individual raw |*S*_12_|^2^(*y*, *f*; *x*_0_) spectra for the feeding source positions 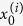
(where *i* = 1, …6) that are evenly spaced inside the 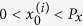 interval. In the case of TTGWs, similar procedure of averaging over the 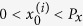 interval is used, but with *i* = 1, 2, 3. The averaging domain spans from 4th rod to 6th rod along the interfaces for both TPSWs and TTGWs cases. The phases plotted in Fig. 5 are extracted from the complex-valued 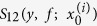 parameters are taken directly from the raw spectra without any spatial averaging (i.e. *i* = 1). Note that the time delays for the TTGWs can be negative around resonances of the detour^20^.

### Edge Scan Measurements

**Figure 1:**
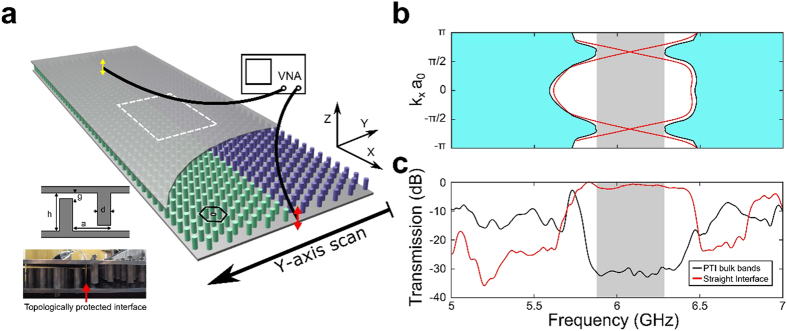
The platform for a quantum spin-Hall photonic topological insulator (QSH PTI): a bianisotropic metawaveguide. **(a)** Schematic of a QSH PTI. Part of the top metal plate is removed to reveal the cylinders attached to the top plate (purple) and to the bottom plate (green), leaving a gap of thickness *g* to the other plate. The 2-port VNA is connected to the feeding source (yellow double arrow) and to the receiving probe (red double arrow) for transmission measurement. Top inset: geometric parameters of the PTI. Bottom inset: picture of the assembled structure showing an interface between the two the PTIs which serve as two topological claddings. **(b)** Calculated 1D projected PBS of the PTI with topologically non-trivial interface. Cyan area: bulk bands, gray area: a complete band gap around the doubly degenerate Dirac cones, red curve: TPSWs supported by the topologically non-trivial interface. **(c)** Measured transmission spectra through the bulk PTI (all rods attached to the top plate: black curve) and along the interface between two PTIs shown in Fig. 1a (red curve). The transmission is enhanced by nearly 30dB in the 5.87 < *f* < 6.29 GHz frequency range by the presence of the interface, indicating that the surface propagation dominates over the bulk PTI propagation. QSH PTI parameters defined in the inset: *h* = *a* = 36.8 mm, *d* = 0.345*a* = 12.7 mm, *g* = 0.15*a* = 5.5 mm.

**Figure 2:**
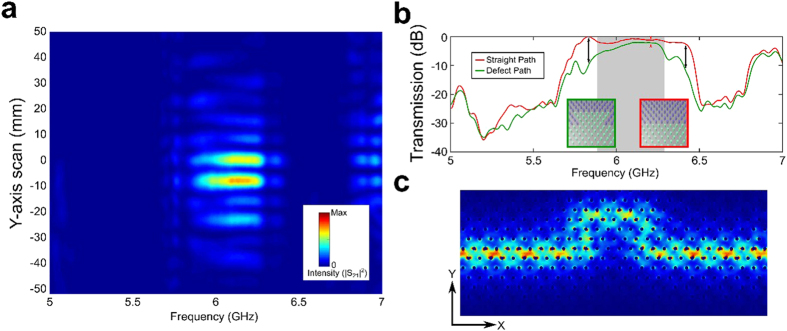
Spatial localization and topological protection of the surface waves. **(a)** Frequency-dependent spatial profiles of the electric field intensity measured at the end of the photonic structure. The surface mode is transversely confined to a small fraction of its wavelength. The zero of the scanning axis (*y* = 0) is at the interface between the two PTIs. **(b)** Comparison of measured transmission spectra between the straight (red curve) and the delay line (green curve) interfaces. The receiving probe is at *y* = 0. Inset: schematic of the interface inside the dashed white box in Fig. 1a for the straight interface (red box) and the delay line interface (green box) containing a large detour-type defect. **(c)** Simulated energy density at *f* = 6.08 GHz showing the TPSW flowing around the defect without scattering.

**Figure 3:**
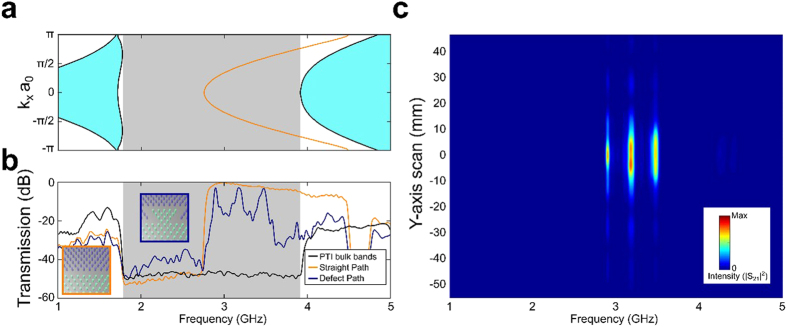
Properties of the guided modes of a topologically trivial waveguide produced by removing one row of rods. The waveguide shown in the orange box in the inset of (b) is classified as topologically trivial because the bulk modes of the *claddings* possess a vanishing Chern index. The center frequency 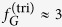 GHz of the topologically trivial bandgap is considerably lower than that of the topologically nontrivial bandgap 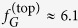 GHz shown in Fig. 1. (**a**) Calculated 1D projected PBS: bulk bands of the claddings (cyan area), a complete band gap (gray area), and the dispersion curve of the TTGW (orange lines). **(b)** Orange box inset: straight waveguide, blue box inset: bent waveguide. Black curve: transmission spectrum through the bulk of the *claddings* (i.e. a row of rods is not removed). Transmission drop in the 1.8 < *f* < 3.9 GHz range indicates the complete bandgap. Near-perfect spectrally-flat transmission (orange curve) through the straight waveguide indicates reveals TTGWs in the 3.0 < *f* < 3.9 GHz range. Transmission through the bent waveguide (blue curve) is negligible for all frequencies except at 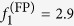 GHz, 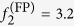 GHz, and 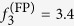 GHz corresponding to Fabry-Perot resonances of the defect. High transmission at 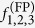 is enabled by multiple bounces of the TTGW inside the defect. **(c)** Frequency-dependent spatial profiles of the electric field intensity measured at the end of the photonic structure. Spatially confined guided modes are observed in three narrow frequency bands.

**Figure 5:**
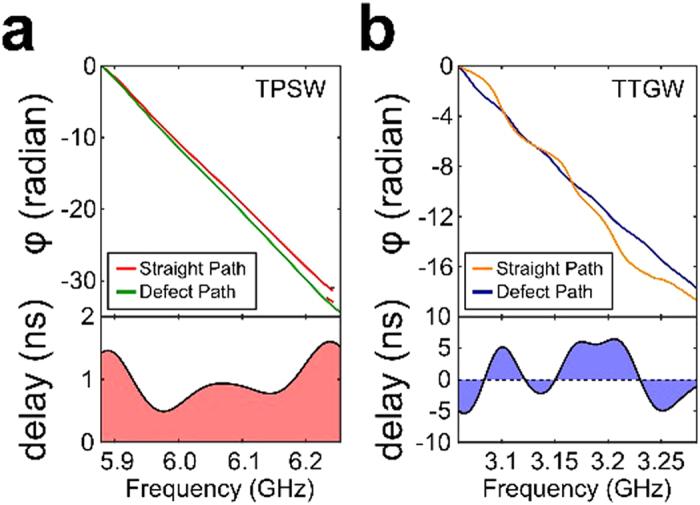
Photonic delay line applications based on QSH PTI. Top half: the relative phases extracted from the transmission spectra of straight path and defect; bottom half: the time delays of delay line application on the platforms of **(a)** TPSWs and **(b)** TTGWs. Note that the time delays for the TTGWs can be negative around the Fabry-Perot (FP) resonances of the detour^20^. Negative time delays are not observed for the detoured TPSWs because topological protection against backscattering prevents FP resonances.

The entire fabricated structure is designed to be 45 periods along the x-direction for long range transport of the surface states, and 20 periods in the y-direction to prevent energy leakage to the lateral boundaries. The receiving probe is placed on the outside at the end of the structure, and can be scanned in the y-direction to map out the transverse profile of the transmitted waves for both the topologically non-trivial waveguide shown in Figs 1 and 2, and the topologically trivial waveguide shown in Fig. 3. The receiving probe is mounted on a motorized stage (VELMEX Single Axis BiSlide) programmed together with the VNA to collect a single *S*_12_(*y*, *f*; *x*_0_) spectrum, and then to reposition the probe by one step Δ*y* = 1.59 mm. Each complete scan consists of *N*_*y*_ = 257 spatial steps totaling *L*_*y*_ = *N*Δ*y* = 406.64 mm span).

### Numerical Simulations

Two types of first-principles frequency domain electromagnetic simulations were performed using the COMSOL package: (a) the eigenvalue simulations which determine the frequencies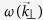, 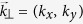 is the in-plane wavenumber, *ω* = 2*πf* is the angular frequency), and (b) the driven simulations that determine the electric/magnetic field distribution for a given current source. To obtain the photonic band structures (PBS) of the topologically trivial/non-trivial waveguides, eigenvalue simulations were carried out on a supercell containing a single period along the x-direction, and 30 cells on each side of the interface. The shaded regions in Figs 1b and 3c are the projected band structure of the bulk modes. The transmission through the photonic structures containing a straight interface and a bent interface with one or two detours were calculated using driven simulations on the simulation domains containing 20 × 45 cells so as to closely approximate the actual structure used in the experiments.

To examine the effect of Ohmic loss on the propagation of the TPSWs at higher frequencies (at *f* = 1 *THz* that corresponds to *λ* = 0.3 *mm*), we performed an eigenfrequency simulation of the proportionally scaled photonic structure. With loss in the metal fully accounted for by an impedance boundary condition at metal surfaces, we found the propagation length to be *L*_*x*_ ≈ 70*a* ≈ 52*λ*, which corresponds to 2.8 dB/cm spatial decay rate. Such propagation distance is sufficiently long for taking advantage of topological protection.

## Additional Information

**How to cite this article**: Lai, K. *et al*. Experimental Realization of a Reflections-Free Compact Delay Line Based on a Photonic Topological Insulator. *Sci. Rep.* **6**, 28453; doi: 10.1038/srep28453 (2016).

## Supplementary Material

Supplementary Information

## Figures and Tables

**Figure 4:**
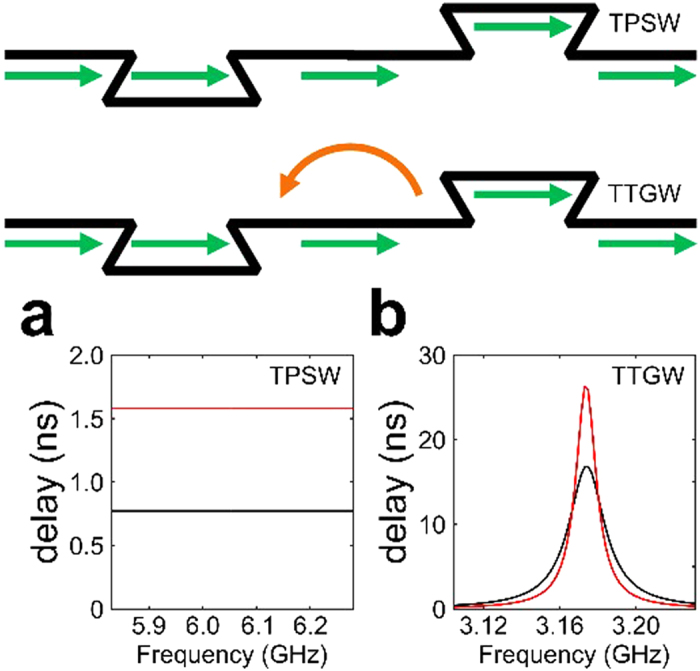
Photonic time delay lines based on detour-type defects. Top: schematics of stacking two defects along the photonic waveguides. The second defect is placed at a distance Δ*x* = 16*a* after the first defect. Green arrows: transmitted, orange arrows: reflected EM waves. **(a)** Two compact detours (red line) produce twice the delay time of the one (black line), with no decrease in the bandwidth for TPSWs. **(b)** In contrast, two detour-type defects in a topologically trivial waveguide reduces the operational bandwidth because of the reflections of TTGWs. While the peak delay is almost doubled by the second defect, the bandwidth is reduced to less than half of the original value. The resultant delay-bandwidth product *N* = Δ*fτ*, which determines the number of electromagnetic pulses that can be buffered by a delay line, does not increase by the addition of the second defect.
