# Supplementary material for: Experimental Realization of a Reflections-Free Compact Delay Line Based on a Photonic Topological Insulator
Source: Sci Rep. 2016 Jun 27;6:28453. doi: 10.1038/srep28453 (PMC4921924; doi:10.1038/srep28453)
Supplement: Supplementary Information [file srep28453-s1.pdf]

Supplementary Information

Experimental Realization of a Reflections-Free Compact Delay Line Based on a Photonic Topological Insulator

Kueifu Lai[1], Tzuhsuang Ma[1], Xiao Bo[2,3], Steven Anlage[2, 3], and Gennady Shvets[1,*]

[1] Department of Physics, The University of Texas at Austin, Austin, Texas 78712, USA

[2] Center for Nanophysics and Advanced Materials, Department of Physics, University of Maryland, College Park, Maryland 20742-4111, USA

[3] Department of Electrical and Computer Engineering, University of Maryland, College Park, Maryland 20742-3285, USA

1. Design and properties of the Quantum Spin Hall Photonic Topological Insulator

The photonic topological insulator (PTI) used in this work is based on the bianisotroic meta-waveguide (BMW) platform [1]. It consists of a hexagonal array of metal rods sandwiched between two parallel metal plates separated by the distance h_0 that confine the electromagnetic waves in the vertical (z-) dimension. When the posts are symmetrically placed between two metal plates (and separated from them by the gap $g_0$as shown in Fig.S1(a)), the structure can be viewed as "photonic graphene" (PhG) [2] with the period a_0 [3]. The PhG structure supports transverse electric (TE) and magnetic (TM) modes shown in Fig.S1(b). The modes differ from each other by their symmetry properties with respect to mid-plane reflection operation σ_z: the $H_z$component of the TE (TM) mode is symmetric (anti-symmetric) with respect to σ_z while the opposite holds for the $E_z$components of the two modes.

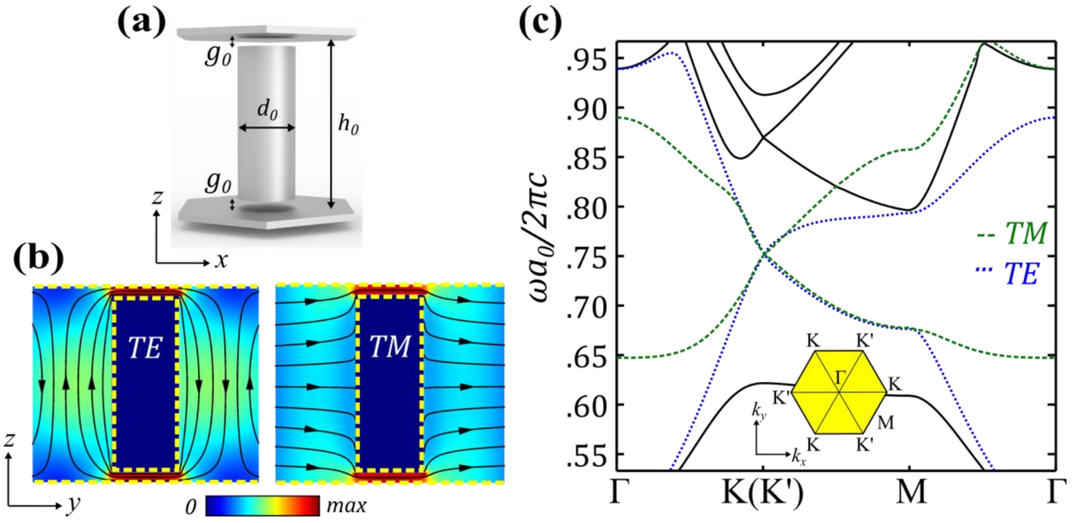

Figure S1: The unperturbed "photonic graphene" (PhG) structure used for emulating photon equivalents of the spin and valley degrees of freedom. **(a)** The unit cell of the PhG: metal rods arranged as a hexagonal array lattice with the lattice constant a_0. **(b)** Magnetic field profiles of the TE and TM modes at the K point. **(c)** The PBS with TE and TM modes forming doubly-degenerate Dirac cones at $K(K')$ points. Design parameters: $h_0 = a_0$, $d_0 = 0.345a_0$, and $g_0 = 0.05a_0$.

Each of the two modes is doubly-degenerate for $\boldsymbol{k}_\perp = \pm \boldsymbol{e}_x 4\pi/3a_0$ corresponding to the $K(K')$ edges of the Brillouin zone shown in the inset to Fig.1S(b). The hexagonal symmetry of the PhG lattice guarantees the appearance of the Dirac cone for the decoupled TE/TM modes. Moreover, for a given period a_0, the two modes can be made degenerate with each other at the $K(K')$ points by the judicious choice of h_0 and the cylinders' diameter d_0. Such mode-degeneracy is essential [4] for establishing spin-like linear combinations of the TE/TM modes which can be coupled to each other by a bi-anisotropic perturbation of the photonic structure. One such perturbation involves simply closing one of the gaps between the rods and the plates, i.e. attaching the rod to one of the plates as shown in Fig.S2. The resulting band gap shown in Fig.S2(b) separates the

photonic phases with the opposite signs of the spin-Chern number $C_{\uparrow/\downarrow}^{SOC} = \pm 1 \times \text{sgn}(\Delta_{SOC})$, where is the bianisotropic coefficient whose sign is determined by the plate to which the metal rod is attached [1]: $\Delta_{SOC} > 0$ if the rod is attached to the bottom plate, and $\Delta_{SOC} < 0$ if the rod is attached to the top plate. The size of the bandgap is also proportional to$|\Delta_{SOC}|$. For those frequencies inside the bandgap, this photonic structure behaves as a photonic topological insulator emulating the quantum spin Hall effect (QSH-PTI). The bianisotropy parameter Δ_{SOC} in PTIs emulates the strength of the spin-orbit interaction in the Kane-Mele model [5] of spin-Hall effect in graphene, which controls the Chern number of the topological phases.

Note that the spin-Chern number is not a global topological index because the sum of the spin-Chern numbers over all spin states vanishes: $\sum_{k=\downarrow,\uparrow} C_k^{SOC} = 0$. However, for the spin-degenerate bianisotropic structures [1] considered here there is no inter-spin scattering. Therefore, two independent copies of the topological phases with opposite spin-Chern numbers can be independently considered.

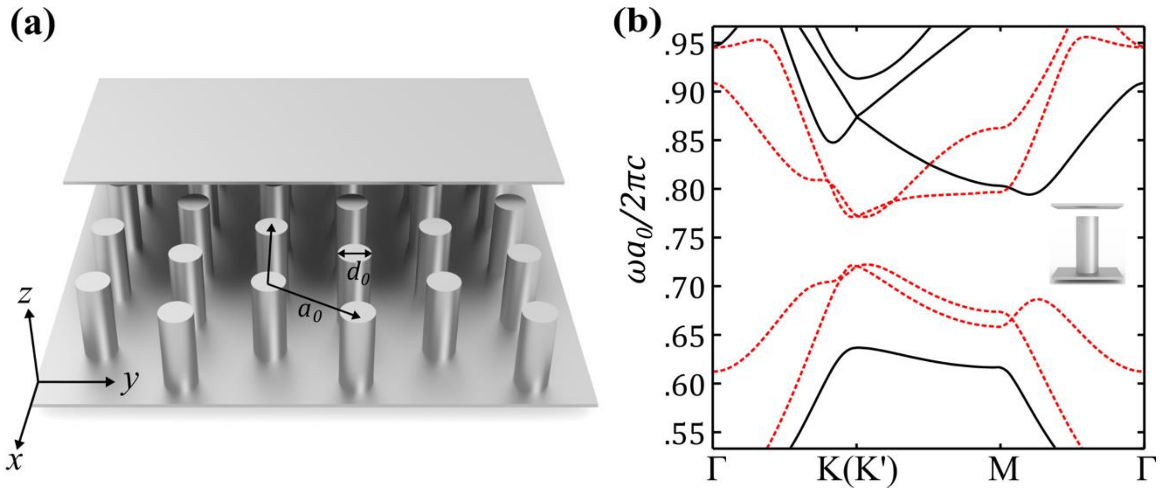

Figure S2: Bi-anisotropic meta-waveguide (BMW) as a photonic topological insulator. (a) Schematic of the BMW. Part of the top metal plate is removed to reveal the "bed-of-nails" structure below. **(b)** PBS with the bandgap induced by the bianisotropy of the meta-waveguide: gap between the rod and the bottom metal plate is closed. Dashed lines: the hybridized TE/TM bands of interest. BMW parameters: $h_0 = a_0$, $d_0 = 0.345a_0$, $g_0 = 0.15a_0$.

The ability to engineer the sign of the Chern number by making a simple change to the structure (e.g., by reversing the orientation of its constituent elements [4] or by attaching the rods to a different plate [1]) is a unique feature of bianisotropic PTIs that are not available with many other designs [6] where the sign of the effective spin-orbit interaction is fixed.

2. Emergence of topologically protected surface waves at the domain wall between two QSH-PTIs

The existence and number of the topologically protected surface waves (TPSWs) at a QSH/QSH interface can be predicted based on the bulk-boundary correspondence principle [7]. One such interface, between a QSH with $\Delta_{SOC}^{(1)} > 0$ and a QSH with $\Delta_{SOC}^{(2)} < 0$, is shown in Fig.S3(a) a thin dashed line. All together there two TPEWs moving in a given direction because $\Delta C = C_{\uparrow(\downarrow)}^{(1)} - C_{\uparrow(\downarrow)}^{(2)} = \pm 2$. Therefore, spin-up TPSWs are moving to the right and spins-down TPSWs are moving to the left along the interface as predicted by the corresponding photonic band structure shown in Fig.S3(b). The field profiles of the four TPSWs labeled by their spins and refractive indices are shown in Fig.S3(c,d) [3].

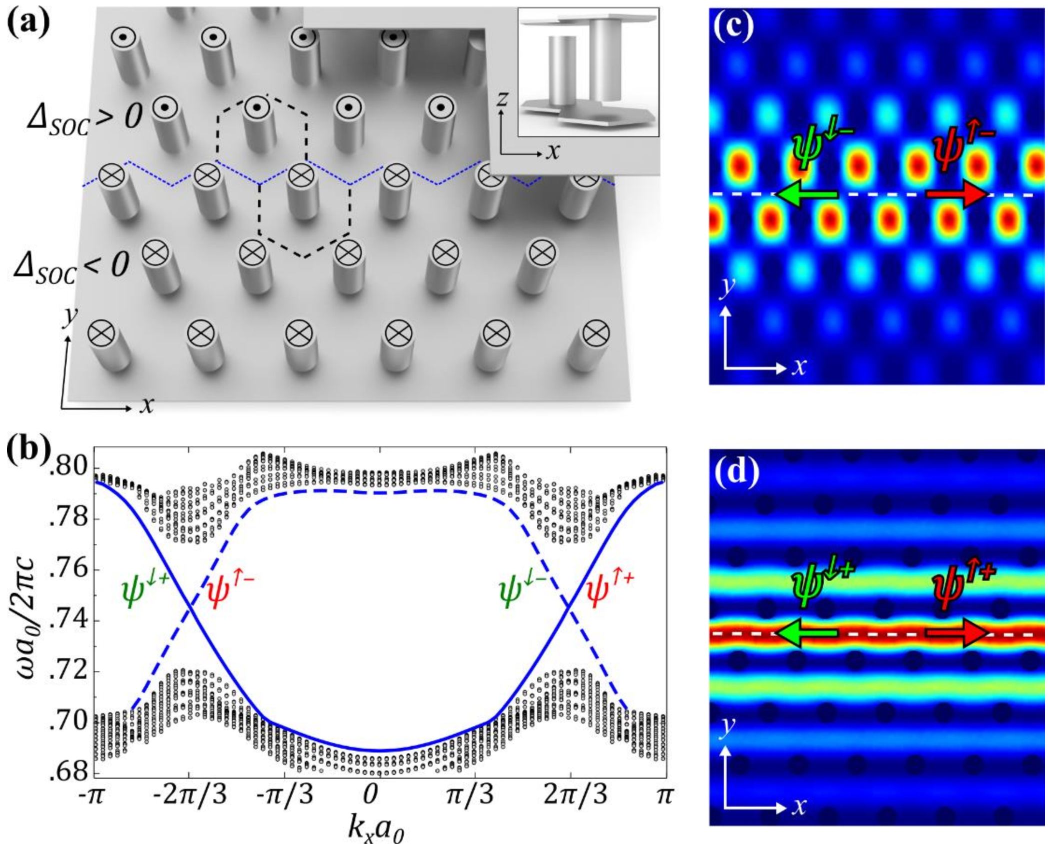

Figure S3: Topologically protected edge modes along QSH/QSH zigzag interface. (a) Top views of the zigzag interface (thin dashed line) between two QSH PTIs investigated in this Letter. Thick dashed line outlines the side view (inset) of two adjacent unit cells on the edge. Black arrows on rods indicate $\mathrm{sgn}(\Delta_{SOC})$. **(b)** PBS of a supercell (single cell along x-direction, 30 cells on each side of the interface). Black circles: bulk modes, blue solid/dashed lines: dispersion curves of the TPSWs with positive/negative refractive index. ↑/↓ labels mark the spin, and +/− labels mark the sign of the refractive index $n(\omega) \equiv ck_x/\omega$. **(c,d)** Field profiles of the TPEWs with positive/negative refractive index respectively. Color: $|E_z \pm H_z|^2$ for ↑/↓ respectively. Red and green arrows show the propagation direction of the edge mode with different spins.

It can be observed from Figs.S3(c,d) that the propagation direction of TPSWs is spin-locked. The two types of spin-up forward-propagating TPSWs have the same group velocity but different phase velocities: one has a positive refractive index and another one has a negative one. In the experiment we are simultaneously exciting both forward-propagating TPSWs because we are using a dipole antenna for excitation. A more sophisticated phased antennas array could be used in the future for exciting just one of the two modes. In addition, one could use a far-field excitation of TPSWs using a narrow slit cut through the top plate as explained in Ref. [8]. An electromagnetic wave incident on the slit at an angle $\phi(\omega)$ with respect to the normal (z-) direction that satisfies the phase matching condition with a given TPSW (i.e. $\sin\phi\,(\omega) = n(\omega)$ for a TPSW with the refractive index $n(\omega) = ck(\omega)/\omega$) would strongly couple to that particular TPSW. These two approaches to selective excitation of TPSWs is the subject of our future work.

3. Preservation of spin-degeneracy and spin conservation in the presence of defects

Because the spin degree of freedom in QSH-PTIs is synthetic, the topological protection of TPSWs does not directly follow from time-reversal symmetry as it does in conventional topological insulators. Instead, TPSWs rely on the properties of spin-degeneracy and the conservation of the spin DOF [1, 4]. Only a limited class of defects ensure these properties. One such lattice defect involves random variations of the magnitude of the effective spin-orbit coupling coefficient Δ_{SOC}, which itself is determined by the gap size g between the metal rod and plate. Changing the size of the gap can be viewed as an electromagnetic perturbation that affects the TE and TM modes differently, thus potentially violating spin-degeneracy. Our earlier analytic estimates quantified [1] the strength of thus induced spin coupling and compared it to the strength of the effective spin-orbit coupling Δ_{SOC}. The former was predicted to be much smaller than the latter.

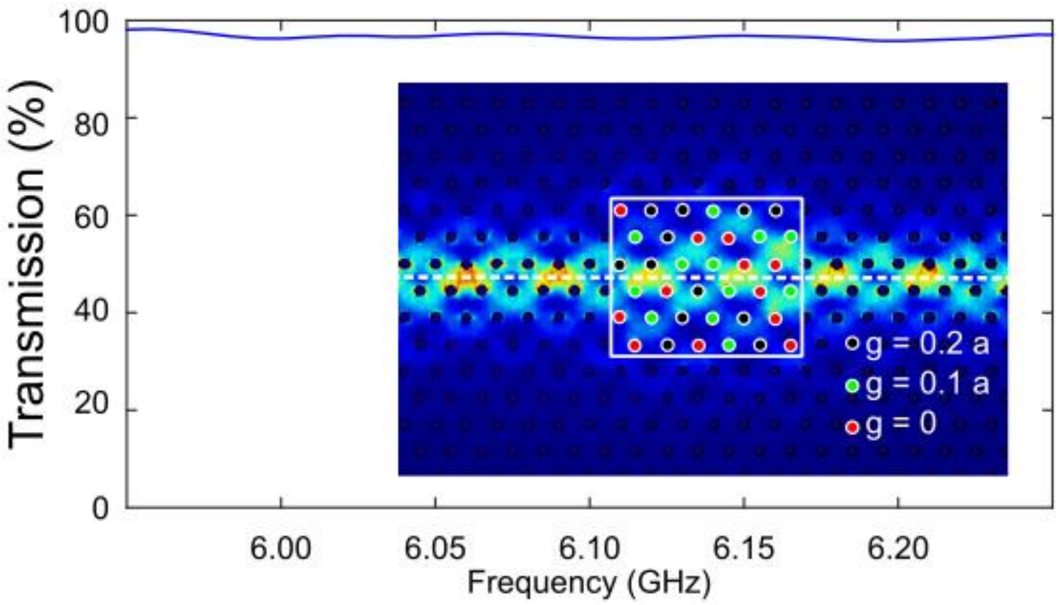

Figure S4| Performance of QSH PTI with Random Gap Sizes. Calculated transmission spectrum of TPSWs propagating along the straight interface through a region of defects with random gap size: $g \neq 0.15a$. Blue Curve: transmission of TPSWs. The transmission is large than 90% over the frequency range of interest. Inset: Energy density of TPSWs propagation. White dashed line: the domain wall between two QSH PTIs (top domain: rods attached to top plate, bottom domain: rod attached to bottom plate). White-bordered box: defect region with color-coded random gap sizes (black: $g = 0.2a$, green: $g = 0.1a$, red: $g = 0$). The TPSWs are excited by a point dipole to the left of the shown domain. Parameters of the QSH PTIs are the same as in the caption to Fig.1 everywhere, except inside the white-bordered box.

To investigate the implications of this near-conservation of the spin DOF, we have devised a numerical experiment shown in Fig.S4. The numerical experiment uses the same sizes (listed in the caption of Fig.1 of the Letter) as the experiment, except that a very large portion of the photonic lattice is perturbed. The perturbed region inside the white-bordered box in Fig.S4 consists of 35 rods ($\approx 4\lambda \times 4\lambda$) near the domain wall

between the two PTIs with opposite values of Δ_{SOC} has been subjected to strong and random perturbation of the gap size g: the baseline value $g = 0.15a$, the varied gap is $0 < g < 0.2a$. Within our computational accuracy, such strong defect does not reduce transmission and does not cause any detectable back-scattering of the TPSWs. That implies that there is no mixing of the spin states by the perturbation and, therefore, no violation of the topological protection. Note that this perturbation does not involve any displacement of the rods from their positions on the hexagonal lattice.

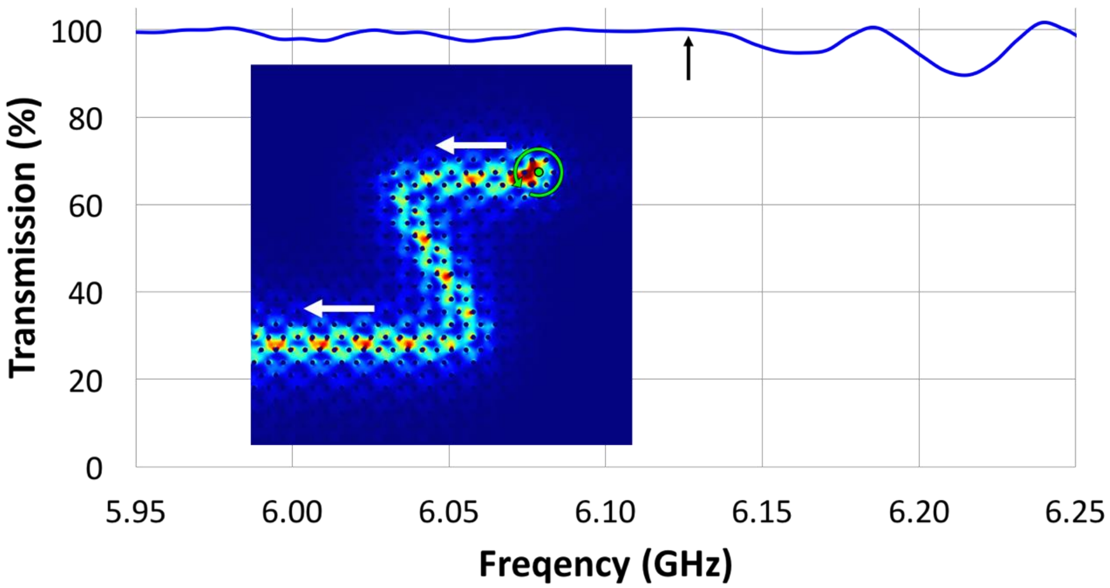

Figure S5: Propagation of TPSWs along the domain wall between two QSH PTIs with opposite signs of the effective spin-orbit coupling. Transmission spectrum $T(\Delta\omega)$ through the zigzag-type path, where $\Delta\omega = \omega - \omega_G$ is the detuning from the bandgap center at$\omega_G a_0/2\pi c = 0.745$. The spin-down TPSWs are excited by placing an electric dipole between the rod and metal plate in the upper right corner. Parameters of the QSH PTIs are the same as in the caption to Fig.1.

The second defect type is the one which is the subject of this Letter: a deformation of the domain wall between the two PTIs. That is, one can design an arbitrarily-shaped domain wall with multiple sharp bends of the propagation path, and the guided surface wave will still propagate through without any backscattering. One such defect, which is a combination of multiple sharp turns of the domain wall, has been theoretically for the case of the two $120°$ zigzag-type bends analyzed [1]. These simulations results are reproduced in Fig.S5.

References

1. Ma, T., Khanikaev, A. B., Mousavi, S. H. & Shvets, G., Guiding Electromagnetic Waves around Sharp Corners: Topologically Protected Photonic Transport in Metawaveguides. *Phys. Rev. Lett.* **114** (12), 127401 (2015).

2. Szameit, A., Rechtsman, M. C., Bahat-Treidel, O. & Segev, M., PT-symmetry in honeycomb photonic lattices. *Phys. Rev. A* **84**, 021806(R) (2011).

3. Ma, T. & Shvets, G., Scattering-Free Optical Edge States between Heterogeneous Photonic Topological Insulators. *arXiv preprint arXiv:1507.05256* (2015).

4. Khanikaev, A. B., Mousavi, S. H., Tse, W.-K., Kargarian, M., MacDonald, A. H. & Shvets, G., Photonic topological insulators. *Nature Materials* **12** (3), 233-239 (2013).

5. Kane, C. L. & Mele, E. J., Quantum Spin Hall Effect in Graphene. *Phys. Rev. Lett.* **95**, 226801 (2005).

6. Chen, W.-J., Jiang, S.-J., Chen, X.-D., Zhu, B., Zhou, L., Dong, J.-W. & Chan, C. T., Experimental realization of photonic topological insulator in a uniaxial metacrystal waveguide. *Nature Communications* **5** (2014).

7. Mong, R. S. & Shivamoggi, V., Edge states and the bulk-boundary correspondence in Dirac Hamiltonians. *Physical Review B* **83** (12), 125109 (2011).

8. Karl, N. J., McKinney, R. W., Monnai, Y., Mendis, R. & Mittleman, D. M., Frequency-division multiplexing in the terahertz range using a leaky-wave antenna. *Nature Photonics* **9**, 717 (2015).
